# Evaluation of Biosynthetic Pathway and Engineered Biosynthesis of Alkaloids

**DOI:** 10.3390/molecules21081078

**Published:** 2016-08-18

**Authors:** Shinji Kishimoto, Michio Sato, Yuta Tsunematsu, Kenji Watanabe

**Affiliations:** Department of Pharmaceutical Sciences, University of Shizuoka, Shizuoka 422-8526, Japan; kishimoto@u-shizuoka-ken.ac.jp (S.K.); michio@u-shizuoka-ken.ac.jp (M.S.); tsunematsu@u-shizuoka-ken.ac.jp (Y.T.)

**Keywords:** alkaloids, biosynthesis, enzymes, engineered biosynthesis, aromatic amino acids

## Abstract

Varieties of alkaloids are known to be produced by various organisms, including bacteria, fungi and plants, as secondary metabolites that exhibit useful bioactivities. However, understanding of how those metabolites are biosynthesized still remains limited, because most of these compounds are isolated from plants and at a trace level of production. In this review, we focus on recent efforts in identifying the genes responsible for the biosynthesis of those nitrogen-containing natural products and elucidating the mechanisms involved in the biosynthetic processes. The alkaloids discussed in this review are ditryptophenaline (dimeric diketopiperazine alkaloid), saframycin (tetrahydroisoquinoline alkaloid), strictosidine (monoterpene indole alkaloid), ergotamine (ergot alkaloid) and opiates (benzylisoquinoline and morphinan alkaloid). This review also discusses the engineered biosynthesis of these compounds, primarily through heterologous reconstitution of target biosynthetic pathways in suitable hosts, such as *Escherichia coli*, *Saccharomyces cerevisiae* and *Aspergillus nidulans*. Those heterologous biosynthetic systems can be used to confirm the functions of the isolated genes, economically scale up the production of the alkaloids for commercial distributions and engineer the biosynthetic pathways to produce valuable analogs of the alkaloids. In particular, extensive involvement of oxidation reactions catalyzed by oxidoreductases, such as cytochrome P450s, during the secondary metabolite biosynthesis is discussed in details.

## 1. Introduction

**Figure 1:**
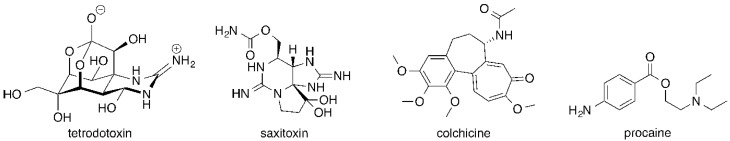
Chemical structures of alkaloids in a broad sense: natural alkaloids of non-plant origin, tetrodotoxin and saxitoxin; a natural but non-basic alkaloid, colchicine; and a synthetic alkaloid, procaine.

Alkaloids were originally defined as organic compounds of plant origin that possess strong bioactivities and exhibit basicity that is attributed to the presence of nitrogen [1]. However, harmful nitrogen-containing compounds, such as tetrodotoxin and saxitoxin (Figure 1), that are isolated from animal or microorganism sources are frequently categorized as alkaloids recently. Similarly, compounds that do not show basicity due to conversion of an amine into an amide in the molecule as seen in colchicine (Figure 1) are also generally identified as alkaloid-type compounds. Even synthetic compounds, such as procaine (Figure 1), that have similar chemical structures or biological properties to plant alkaloids can be referred to as alkaloids. As such, it has become commonplace nowadays to define alkaloids as nitrogen-containing organic compounds that can produce pronounced physiological responses, with notable exceptions being amino acids, nucleic acids and certain natural nitrogen-containing compounds, such as tetracycline (polyketide) and kanamycin (aminoglycoside). The alkaloid class of natural products has been very important for humanity as a valuable source of pharmaceutical products, because they show a variety of biological activities at a very low dosage. Therefore, alkaloids have been frequently employed as lead compounds for drug discovery. On the other hand, during the past decade, development of new DNA sequencing technologies, in particular next-generation sequencing, has enabled us to easily explore “the master plan of alkaloid production”; that is, the genes responsible for the biosynthesis of alkaloids. By studying the master plan, we started to gain deep insight into “the implementation of the master plan”, meaning the mechanism of the biosynthesis of alkaloids. Furthermore, we are using the gained knowledge to look for new alkaloids and at the same time modify the “master plan” to engineer production of modified natural products. In this article, we focus on five successful examples of reconstitution of alkaloid biosynthetic pathways in heterologous microbial hosts. Those five pathways are responsible for the formation of ditryptophenaline (dimeric diketopiperazine alkaloid), saframycin (tetrahydroisoquinoline alkaloid), strictosidine (monoterpene indole alkaloid), ergotamine (ergot alkaloid) and opiates (benzylisoquinoline and morphinan alkaloid). Discussion of the use of microbes as catalysts for the bioconversion of advanced alkaloid intermediates can be found elsewhere [2]. Similarly, readers are referred to other review articles for the recent efforts toward engineering plants for improved production of alkaloids and their analogs [3,4]. We will also discuss the recent progress in the development of methodology for generating various compounds through heterologous reconstitution and engineering of biosynthesis pathways, especially those involved in the bioproduction of opioids.

## 2. Ditryptophenaline

**Figure 2:**
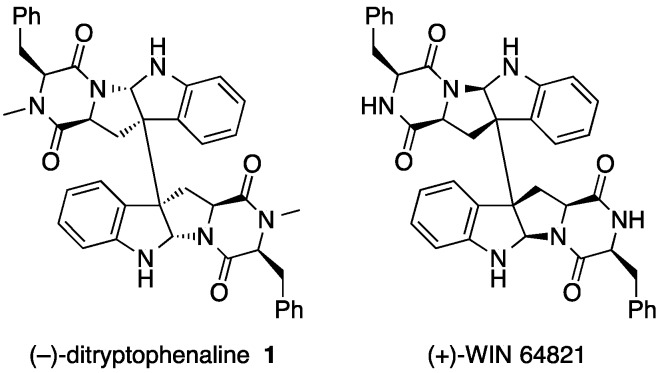
Chemical structures of dimeric diketopiperazine alkaloids, (–)-ditryptophenaline **1** [7] and (+)-WIN 64821 [8].

Ditryptophenaline (**1**) is a dimeric diketopiperazine alkaloid [5] and has a complicated structure (Figure 2). Dimerization is found relatively frequently among natural products, as it is considered to be an efficient way of enhancing the chemical complexity and diversity of metabolites being biosynthesized. It can also increase the valency of a compound, affording the molecule a higher affinity for its target or higher stability upon forming a complex with its target [6]. Compound **1** [7] and its related compound WIN 64821 (Figure 2) [8] originally isolated from *Aspergillus versicolor* exhibit useful biological activities, such as an inhibition of substance P receptor for potential analgesic and anti-inflammatory activities. Despite their complex chemical structures, their formation seemed relatively simple, involving dimerization of diketopiperazine monomers formed through condensation of two amino acids at the initial stage. Judging from the structure of the diketopiperazine monomer of **1**, it appeared that l-tryptophan and l-phenylalanine could be condensed together to form the diketopiperazine core structure of the monomer. Once the phenylalanine-derived amide nitrogen of the diketopiperazine core is *N*-methylated, a ring closure within the monomer and a subsequent monomer–monomer crosslinking through oxidation reactions involving the tryptophan indole ring can lead to the formation of a homodimer of **1**.

**Figure 3:**
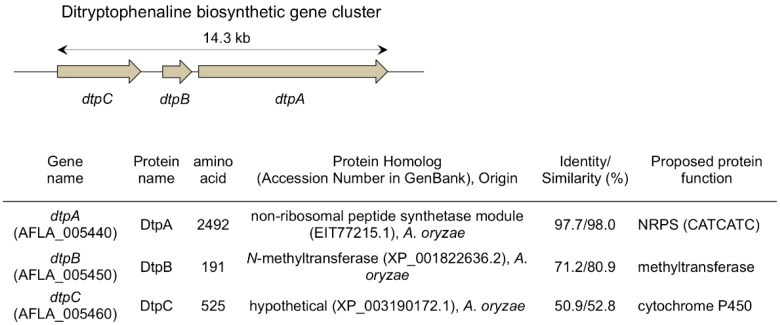
*Aspergillus flavus* ditryptophenaline biosynthetic gene cluster and predicted functions of the genes in the cluster.

**Figure 4:**
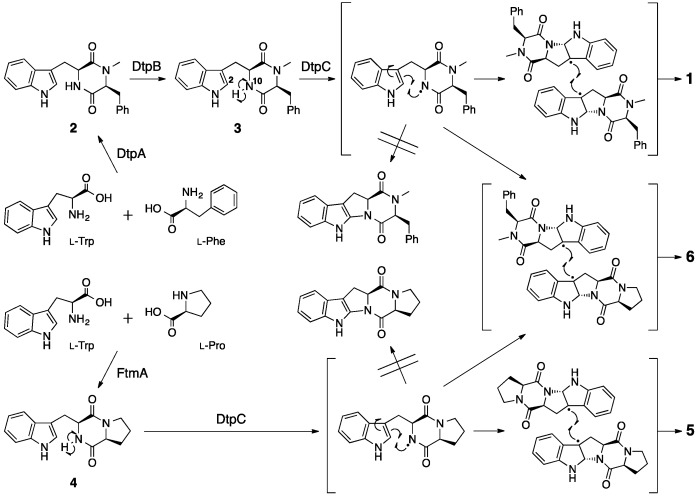
Proposed mechanism of DtpC-catalyzed dimerization of intermediates **3** and **4** to form (−)-ditryptophenaline **1**, (−)-dibrevianamide F **5** and (−)-tryprophenaline **6** via a radical route [9]. DtpA, ditryptophenaline nonribosomal peptide synthetase (NRPS); DtpB, *N*-methyltransferase; DtpC, cytochrome P450; FtmA, fumitremorgin NRPS.

More recently, **1** was found to be produced by another fungus, *Aspergillus flavus* [9]. To elucidate the biosynthetic pathway of **1**, experiments of deficiency of the biosynthetic gene were performed to identify ditryptophenaline biosynthetic gene cluster. Based on the homology of amino acid sequences deduced from the *A. flavus* genome sequence [10] to known enzymes, three genes, *dtpA*, *dtpB* and *dtpC*, clustered together on the *A. flavus* genome were predicted to code for a nonribosomal peptide synthetase (NRPS), an *N*-methyltransferase and a cytochrome P450 (P450), respectively (Figure 3) [9]. Knockout of any one of the three genes in the *A. flavus ∆pyrG*/*∆ku70* strain A1421 [11] resulted in the loss of production of **1**, indicating that these three genes were involved in the biosynthesis of **1**. Based on the in vitro assays of DtpB using a substrate **2** isolated from the *dtpB* knockout mutant, DtpB was shown to catalyze an *N*-methylation of **2** to biosynthesize the intermediate **3**. Interestingly, **3** was directly converted to **1** by DtpC (P450), which was prepared as a microsomal fraction of *Saccharomyces cerevisiae* expressing *dtpC* recombinantly. The analysis of the in vitro experiments suggested that DtpC catalyzes the C2–N10 ring closure within the monomer and the dimerization of the tetracyclic monomers, both of which likely proceed through a radical pathway. In the proposed mechanism (Figure 4), a radical formed at N10 through hydrogen atom abstraction by the heme iron of P450 can migrate to C3 with a concomitant C2–N10 ring closure and undergo a radical-mediated coupling with another C3 radical-bearing monomer to form the bridging bond in the dimer like **1**.

**Figure 5:**
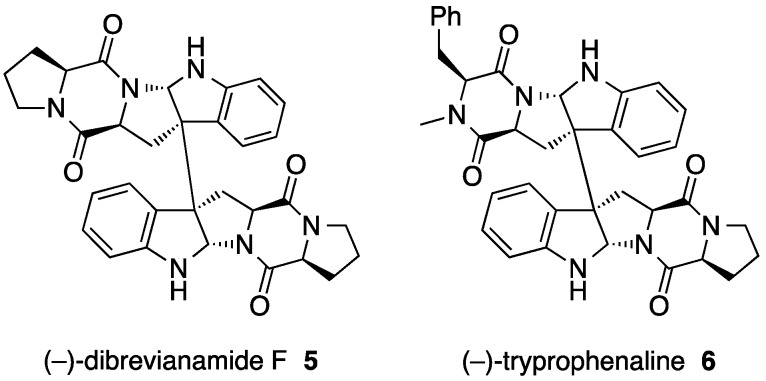
Chemical structures of the newly formed compounds, (–)-dibrevianamide F **5** and (–)-tryprophenaline **6** [9].

**Figure 6:**
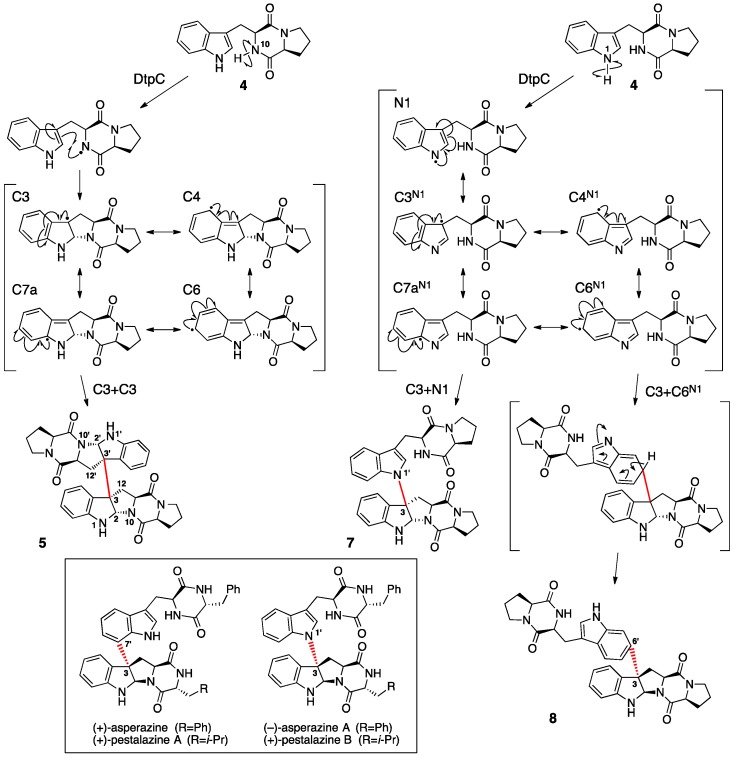
Proposed mechanism of DtpC-catalyzed formation of dimerization products (−)-dibrevianamide F **5** [9], a new compound **7** [14] and naseseazine B **8** [15]. Radical-mediated formation of **7** and **8** is thought to be initiated by DtpC forming a radical at N1 instead of N10 as proposed for the formation of **5**. The radical species are labeled by the position of the radical. To differentiate between radical species derived from hydrogen extraction at N10 (**left column**) vs. N1 (**right column**), those formed via the N1 hydrogen extraction are given a label with a superscript N1. Chemical structures of related known natural products are shown in the box. The monomer-bridging bonds are shown in red.

To examine the substrate tolerance of DtpC and to biosynthesize new dimeric products using DtpC in this study, brevianamide F (**4**) was provided to this enzyme as a substrate [9]. When adding only **4**, compound **5** was produced. Furthermore, adding both the natural substrate **2** and the substrate analog **4** provided compounds **6** as well as **5**. Using NMR and X-ray crystallographic data on **5** and **6**, the chemical structures of these compounds, including their absolute configurations, were determined. The homodimeric product **5** was named (−)-dibrevianamide F, and the heterodimeric product **6** was named (−)-tryprophenaline as new alkaloids (Figure 5). As in **1**, the stereochemistry of chiral carbon atoms in **5** and **6** was determined to be all in the *S* configuration by the Flack parameter value obtained from the crystallographic data. Thus, the biosynthetic mechanism for the formation of **5** and **6** is likely the same as that described above for the formation of **1** (Figure 4). Furthermore, as an example of engineered biosynthesis of non-native dimeric products, the heterologous host *Aspergillus niger* A1179 modified [12] with the introduction of *ftmA* (fumitremorgin NRPS [13]) genes was provided with the *dtpC* gene [14]. This engineered strain was able to isolate additional dimeric compounds **7** and **8** (Figure 6). These two compounds were analogs of the homodimeric product **5** previously produced (Figure 4). Elucidation of the chemical structures **7** and **8** showed **7** to be a new compound and **8** to be naseseazine B isolated recently from a marine-dwelling streptomycete bacterium [15]. Dimerization in **7** and **8** did not form the C3–C3’ linkage found in **1**, **5** and **6**. In **7**, dimerization occurred between C3 and N1’, whereas positions C3 and C6 were involved in the dimerization that formed **8**. Those dimeric compounds are speculated also as products of a radical-mediated oxidative dimerization of tryptophan derivatives, where the linkages are formed at various different positions. DtpC is thought to extract the hydrogen from the diketopiperazine core amide nitrogen N10 of **2** to yield a radical. Then, the radical can migrate to C3 through the C2–N10 ring closure, and the subsequent coupling can proceed to form the C3–C3’ linkage, giving the dimeric product **1**. However, when DtpC was provided with the unnatural substrate **4**, which lacks the bulky phenyl side chain group, the positioning of **4** within the active site of DtpC may be shifted from where **2** would be bound. This may cause an occasional extraction of the hydrogen from the indole nitrogen to yield a radical at N1, which can start migrating from the indole nitrogen to C6 and back to N1 (Figure 6, right column). Coupling of the radical at C3 in one monomer formed via the N10 extraction pathway (labeled as C3 in Figure 6, left column) with the radical at N1 in the other monomer formed via the N1 extraction pathway (labeled as N1 in Figure 6, right column) can produce the dimeric compound **7**. Similarly, coupling of the same C3 radical in one monomer with the C6 radical in the other monomer (labeled as C6^N1^ in Figure 6, right column) can produce **8**. The titer of these compounds was significantly lower than that of the native product **1** (approximately 350 µg/L of culture for **7** and **8** as compared to 2.3 mg/L of culture for **1**) [9,14], presumably because of the low frequency of the N1 hydrogen extraction happening inside the active site of DtpC. There are other known natural products having similar dimerization patterns. For example, (–)-asperazine A [16] and (+)-pestalazine B [17,18] have the same C3–N1 linkage as **7**, whereas (+)-asperazine [19] and (+)-pestalazine A [17,20] have a C3–C7 linkage that was not observed in our study (Figure 6, boxed). Presumably, the biosynthesis of those dimeric diketopiperazine compounds is also catalyzed by P450 enzymes with relaxed substrate specificity, much like DtpC.

## 3. Saframycin

**Figure 7:**
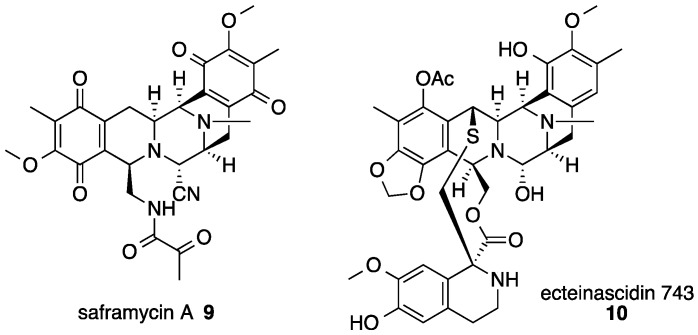
Chemical structures of tetrahydroisoquinoline alkaloids, saframycin A **9** [21] and ecteinascidin 743 **10** [24].

Saframycin A (**9**) isolated from *Streptomyces lavendulae* is a representative member of the tetrahydroisoquinoline alkaloids (Figure 7) [21]. This class of natural products has been isolated from various organisms, including soil bacteria and marine invertebrates, such as sponges and ascidians [22]. These compounds exhibit various antibiotic activities but also possess a potent antitumor activity, which is thought to be effected through covalent modification of DNA by iminium ions via carbinolamine moiety and its equivalents [23]. Among the saframycin analogs, ecteinascidin 743 (**10** in Figure 7) [24] showed a remarkably potent antitumor activity against not only common tumor cell lines but also resistant cell lines [25]. Recently, **10** was approved as an anticancer drug in the European Union for patients with soft tissue sarcoma [26]. Because of the limited supply from natural sources, **10** is currently produced by a semisynthetic process [27]. However, this synthesis requires more than 25 chemical transformation steps, making it a suboptimal method for a commercial production of **10**. Engineered biosynthesis for generating this important alkaloid as an alternative strategy is strongly desired, especially from the perspective of sustainability and environmental burden of the synthetic manufacturing process. To put this bioproduction approach into practice, detailed understanding of the biosynthetic mechanism of this class of natural products is essential. Therefore, there has been a considerable study on the subject involving gene isolation [28,29,30], substrate feeding experiments [31,32], biochemical characterizations [30,33] and bioinformatic analyses [30]. In this review, the current understanding of the mechanism of biosynthesis of **9** is described.

Investigation of the biosynthesis of **9** and related compounds has been ongoing for quite some time after the initial discovery of **9** in 1977 [21]. By 1985, it was determined that the main framework of **9** was comprised of one molecule each of L-alanine and glycine and two molecules of l-tyrosine [31]. The alanyl-glycyl terminal moiety was found to be modifiable by feeding of particular amino acids, such as 2-amino-*n*-butylic acid [32]. The initial discovery of a saframycin biosynthetic gene cluster was made in *Myxococcus xanthus*, where two NRPS-encoding genes and an *O*-methyltransferase gene responsible for the formation of the core structure were identified [28]. The findings from this study confirmed that the main framework of **9** was biosynthesized by NRPS. The subsequent study on the gene cluster for the biosynthesis of safracin, a compound closely related to **9**, revealed additional genes proposed to be involved in the modification of building block amino acids and post-NRPS intermediates [29]. The most recent study provided the most complete information on the saframycin biosynthetic gene cluster with a total of 30 genes that are proposed to be involved in the biosynthetic process [30].

**Figure 8:**
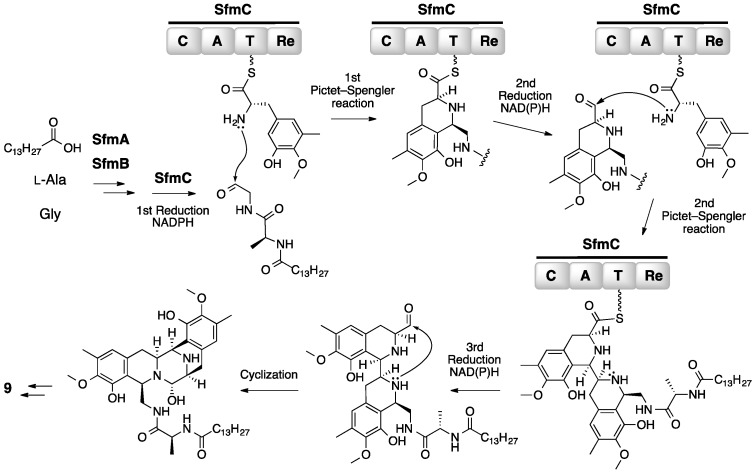
Proposed overall scheme for the pentacyclic core skeleton assembly during the biosynthesis of saframycin A **9** [35]. SfmA, SfmB and SfmC are three saframycin A nonribosomal peptide synthetases. C, condensation domain; A, adenylation domain; T, thiolation domain; Re, reductase domain.

Based on the bioinformatically predicted functions of the genes that were identified as described above, various biosynthetic schemes were hypothesized [29,31,34]. However, no actual experimental data were available to verify the proposed mechanisms. Thus, to establish the mechanism of how the complex core structure of **9** was biosynthesized, in vitro assays on the recombinantly prepared SfmA and SfmB were carried out [30]. As predicted from the adenylation domain amino acid sequence [30], two NRPSs, SfmA and SfmB, were shown to specifically activate l-alanine and glycine, respectively, indicating that those two enzymes were responsible for the formation of the non-tyrosine dipeptide segment of **9** (Figure 8). However, amino acid sequence analysis of SfmA also revealed the presence of an *N*-terminal acyl-CoA ligase (AL)-acyl carrier protein (ACP) didomain [30,35], which is commonly found in NRPSs that form lipopeptides having a long fatty acyl chain extension at the *N*-terminus of the core peptide [36,37]. This finding prompted analysis for the requirement of an *N*-terminal acyl chain in the l-alanyl-glycyl intermediate. In vitro assays with synthetically prepared l-alanyl-glycyl-*S*-CoA substrates having an *N*-acyl chain of various length and SfmC that was recombinantly prepared in *Escherichia coli* clearly indicated that the *N*-myristoylated dipeptide was the actual substrate of SfmC [35]. No reaction took place when substrates lacking a long acyl extension were provided to SfmC. Because the final product **9** does not carry any acyl chain, the requirement of SfmC for a cryptic acyl chain in its substrate is a completely unexpected finding.

Once the actual substrate for SfmC was identified, the mechanism of the formation of the pentacyclic tetrahydroisoquinoline core was investigated. Further detailed examinations, which involved the use of additional synthetic substrates and SfmC mutants [35], demonstrated that SfmC was solely responsible for the formation of the pentacyclic scaffold from two molecules of a l-tyrosine derivative, 3-hydroxy-5-methyl-*O*-methyltyrosine (Figure 8) [30]. Based on the results from those experiments, the reaction mechanism was suggested with the Re (reduction) domain of SfmC catalyzing three reduction reactions and the C (condensation) domain catalyzing two cycles of the Pictet-Spengler reaction to form the tetrahydroisoquinoline core of **9** [35]. The last cyclization reaction after the third reduction step may also be catalyzed by SfmC, although the reaction could proceed spontaneously. While further investigations into the biosynthesis of **9** and related compounds are required, elucidation of this complex reaction mechanism would provide us with a novel chemoenzymatic approach toward synthesizing sufficient amount of **10** and its analogs.

## 4. Strictosidine

Terpene indole alkaloids (TIAs) constitute one of the largest and most diverse families of complex nitrogen-containing metabolites found in plants [38,39]. Many TIAs, especially monoterpene indole alkaloids (MIAs) and their analogs, have medicinal properties: camptothecin and vinblastine are approved as anticancer therapeutics, whereas reserpine is used as an antihypertensive [40]. Physostigmine is an antidote of anticholinergic toxicity and is used primarily for treating glaucoma [41]. However, these structurally complex compounds can be difficult to synthesize chemically [42,43]. To supply these compounds through biosynthetic routes, the biosynthetic pathway for a target compound must be elucidated. Once all of the genes involved in the pathway are identified, a host organism that is more convenient to cultivate than a plant can be used for heterologous production of the desired compound. Furthermore, elucidation of the biosynthetic pathway and establishment of a heterologous system would allow engineering of the pathway for efficient analog production. From these viewpoints, the most important compound in this class of natural products to engineer a heterologous production system would be strictosidine (**11**), because it is the universal intermediate for the biosynthesis of all MIAs. Below, the biosynthetic pathway leading to the formation of **11** and recent efforts in the engineered biosynthesis of **11** using a yeast host system are discussed. 

**Figure 9:**
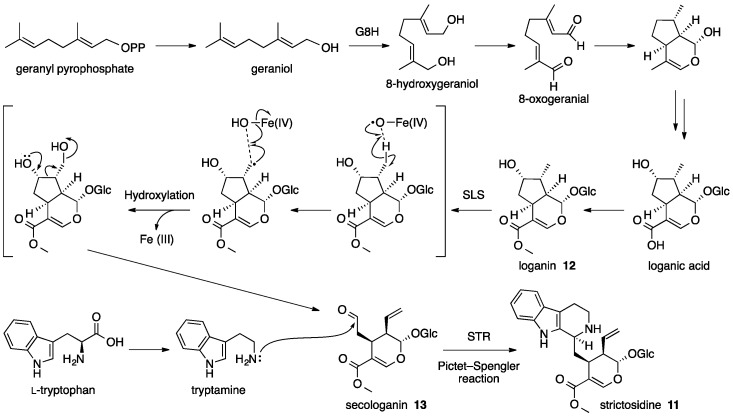
Proposed biosynthetic pathway of strictosidine **11**, the universal intermediate in the monoterpene indole alkaloid biosynthesis [38]. G8H, geraniol 8-hydroxylase; SLS, secologanin synthase; STR, strictosidine synthase.

The general mechanism of the biosynthesis of **11** has been reviewed elsewhere [38]. Briefly, **11** is formed with two starting substrates, geraniol and l-tryptophan. The first starting substrate geraniol is initially converted into a dihydroxy intermediate, 8-hydroxygeraniol, by geraniol 8-hydroxylase (G8H). Then, both hydroxyl groups are oxidized to form 8-oxogeranial. Subsequent cyclization and oxidation of 8-oxogeranial yields loganic acid, and methylation of the carboxylic acid moiety of loganic acid results in the formation of loganin (**12**) (Figure 9). The following transformation of **12** into secologanin (**13**) is an interesting reaction that is catalyzed by secologanin synthase (SLS, a cytochrome P450). This step is thought to be the rate-limiting step in the biosynthetic pathway leading to the formation of **11** [44]. SLS is proposed to catalyze the opening of the 5-membered ring of **12** by a radical-mediated hydroxylation, followed by a dehydration to yield **13** (Figure 9) [45]. The other starting substrate l-tryptophan is modified through a decarboxylation step to afford tryptamine, which is combined with **13** to give **11** by the action of strictosidine synthase (STR, an amine lyase), a key enzyme in MIA biosynthesis. This transformation would be the most chemically interesting step in the strictosidine biosynthetic pathway; it is an another example of the Pictet-Spengler reaction applied toward the biosynthesis of alkaloids, as it was also mentioned during the discussion of the saframycin biosynthesis, (Figure 9). As to the formation of **11**, the reaction is proposed to occur between the aldehyde of **13** and the amine of tryptamine to couple the two substrates through the formation of a β-carboline moiety [46].

To develop a yeast strain that can biosynthesize **11** de novo, 21 gene additions and three gene deletions were introduced into the yeast genome [47]. Fourteen of the added genes were known genes from a MIA producer plant *Catharanthus roseus* [48]. Of the 14 genes, 11 constituted the actual strictosidine biosynthesis pathway and the other three were involved in enhancing the activity of P450s. Among the remaining seven added genes, five were existing yeast genes that were included as an extra copy of the genes in the genome. Incorporation of multiple copies of genes is frequently performed in building a heterologous production system to increase the expression level of specific genes for improved productivity of the biosynthetic pathway of interest [49]. This strategy was also applied to the strictosidine biosynthetic gene *G8H*, where four copies of the codon-optimized *G8H* gene were incorporated into the yeast genome to allow production of **11** without exogenous feeding of geraniol. Lastly, two more genes, *AgGPPS2* and *mFPS144*, were taken from exogenous sources and incorporated into the yeast genome to increase the cellular pool of geranyl pyrophosphate (GPP). AgGPPS2 is a GPP synthase from grand fir (*Abies grandis*) that is known to produce only GPP and does not convert GPP further into farnesyl pyrophosphate (FPP) [50], and mFPS144 is an N114W mutant of avian FPP synthase from the red junglefowl (*Gallus gallus*) that produces GPP nearly exclusively [51]. Those two genes were used to replace the yeast FPP synthase gene *ERG20*, which was one of the three yeast genes deleted from the genome for the biosynthesis of **11**. The other two genes were *ATF1* and *OYE2*, both of which are involved in metabolizing geraniol into other products. After those extensive modifications, the engineered yeast successfully produced **11** at a yield of approximately 0.5 mg/L of culture medium comprised of only simple carbon and nitrogen sources [47]. This yeast strain provides an important first step toward building a microbial system for the production of expensive, complex molecules that only plants produce in small amounts.

## 5. Ergotamine

**Figure 10:**
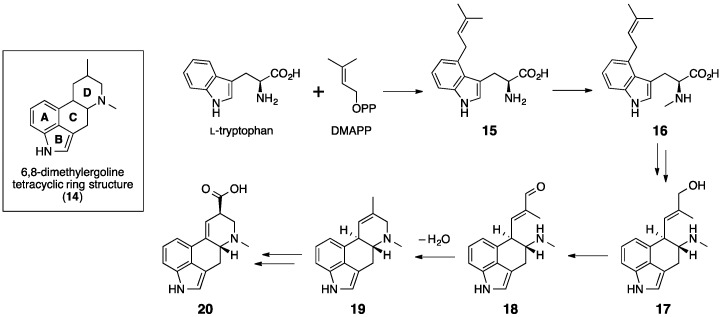
Proposed biosynthetic pathway of d-lysergic acid **20** [53], having the principal 6,8-dimethylergoline tetracyclic ring structure **14** of ergot alkaloids.

Ergot alkaloids form another family of alkaloids under the structurally diverse group of TIAs. Unlike MIAs described in the previous section that use GPP and l-tryptophan as their building blocks, ergot alkaloids are derived from dimethylallyl pyrophosphate (DMAPP) and l-tryptophan [52]. The ergot type of natural products exerts biological activities by acting as either an agonist or an antagonist toward various receptors for a number of neurotransmitters, such as dopamine, serotonin, adrenaline and noradrenaline [53]. Certain members of ergot alkaloids have also been shown to exhibit anti-tumor activities [54]. The potent bioactivities of ergot alkaloids have led people to use them in various medical applications since early in the human history, with the oldest record dating back to around 1000 B.C.E. [55]. The ergot alkaloids are produced by fungi and plants, but the Clavicipitaceae family of fungi is most well known for ergot production. Among the Clavicipitaceae family, *Claviceps purpurea*, an endophytic parasite that is also known as the ergot fungus of rye, and *Neotyphodium lolii*, an endophytic symbiont, are the two most-studied ergot-producing fungi [52]. Another fungus known for production of the ergot family of natural products, primarily clavine-type alkaloids, is *Aspergillus fumigatus*, an opportunistic pathogen of mammals [56]. Below, we will focus on the biosynthetic pathway of ergot alkaloids, and in particular discuss the mechanism involved in the formation of the interesting backbone core of 6,8-dimethylergoline tetracyclic ring structure (**14**) that is shared by all ergot alkaloids (Figure 10).

Biosynthesis of ergot alkaloids was initially investigated through feeding experiments that used isotopically labeled substrates in the cultures of *C. purpurea* [53]. Based on those experiments, the ergot biosynthetic pathway was determined to be initiated by the prenylation of l-tryptophan with DMAPP that forms 4-(γ,γ-dimethylallyl) tryptophan (**15**) (Figure 10) [57,58]. The next step involves the *N*-methylation of **15** to yield 4-dimethyl-l-abrine (**16**) [59]. Subsequently, a series of successive oxidation steps is proposed to take place, resulting in the intramolecular cyclization of the prenyl and indole moieties to form the tricyclic chanoclavine-I (**17**) along with ring C of the tetracyclic core of ergot alkaloids [60]. Compound 17 is then oxidized to form chanoclavine-I-aldehyde (**18**) from which ergot alkaloid biosynthesis can diverge. In one pathway, 18 can undergo intramolecular cyclization to form ring D to complete the tetracyclic ergoline core structure, yielding agroclavine (**19**). Subsequently, **19** is converted to d-lysergic acid (**20**) via an oxidation of a methyl group as illustrated in Figure 10. In another pathway, **18** undergoes a similar cyclization to form a closely related tetracyclic intermediate, festuclavine, which becomes the core molecule for the clavine-type fumigaclavines and related compounds produced by *A. fumigatus*. In this section, we will focus on the biosynthesis of ergotamine (**21**) from **20**, which takes place in *C. purpurea* and *N. lolii*.

**Figure 11:**
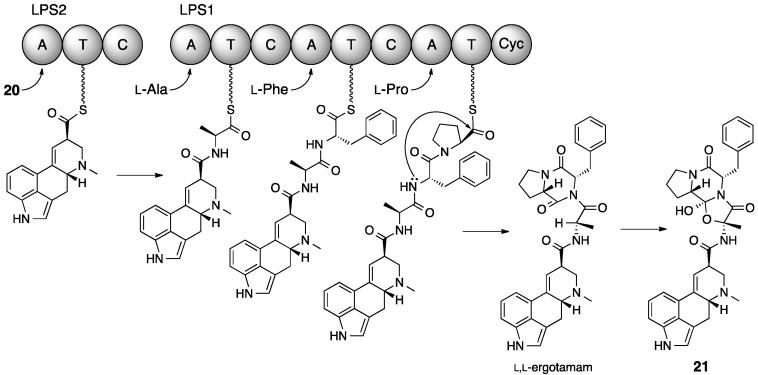
Nonribosomal assembly of the peptide portion of ergotamine **21**, initiated with d-lysergic acid **20** as a primer unit [64]. LPS, d-lysergyl peptide synthetase; C, condensation domain; A, adenylation domain; T, thiolation domain; Cyc, cyclolization domain.

Further steps in the biosynthesis of ergotamine (**21**) have been elucidated predominantly by Keller et al. [61,62,63,64]. Those steps mainly concern the formation and the modification of the peptide-derived segment of **21** (Figure 11). The peptide extension of **20** is initiated by LPS2 (lysergic peptide synthetase **2**, NRPS). The adenylation domain of LPS2 accepts **20** as the starter unit, and the condensation domain couples it with l-alanine, which is activated by the first adenylation domain of LPS1 (lysergic peptide synthetase **1**, NRPS), through an amide linkage formation. The resultant d-lysergyl-monopeptide intermediate is extended further with l-phenylalanine and l-proline by LPS1 to yield the d-lysergyl-tripeptide, which remains tethered to the phosphopantetheinyl arm of the third thiolation domain of LPS1. The terminal cyclization or lactamization domain is thought to catalyze the release of the d-lysergyl-tripeptide from LPS1 as *N*-(d-lysergyl-l-Ala)-l-Phe-l-Pro-lactam (l,l-ergotamam). Finally, l,l-ergotamam is converted into ergotamine (**21**) by the hydroxylation activity of the non-heme dioxygenase EasH (Fe^2+^/2-ketoglutarate-dependent dioxygenase) [64].

Very recently, heterologous partial reconstitution of the ergot alkaloid biosynthetic pathway was accomplished in *Aspergillus nidulans* by introducing a segment of the *A. fumigatus* genome covering a portion of the ergot alkaloid biosynthetic gene cluster into the *A. nidulans* chromosome [65]. The integrated *A. fumigatus* genomic DNA segment contained four genes, dimethylallyltryptophan synthase-coding *dmaW*, dimethylallyltryptophan *N*-methyltransferase-coding *easF*, oxidoreductase-like protein-coding *easE* and catalase-like protein-coding *easC*. The transformed *A. nidulans* was able to produce **16** and **17**, early intermediates in the d-lysergic acid biosynthetic pathway (Figure 10). Using this heterologous production system, gene deletion experiments were performed to determine for the first time that the four genes were sufficient for the biosynthesis of **17** [65]. This successful production of ergot alkaloid biosynthetic intermediates in *A. nidulans* would facilitate the effort toward achieving engineered biosynthesis of this class of natural products. Such a heterologous biosynthetic platform would promote the study on the complex mechanism of ergot alkaloid biosynthesis and advance the drug discovery efforts that evolve around ergot-type natural products.

## 6. Opiates

**Figure 12:**
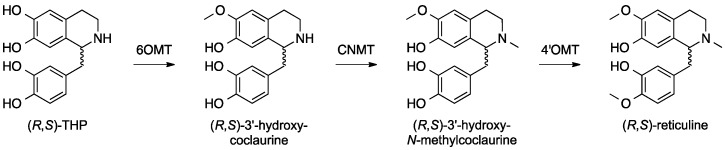
Heterologous biosynthetic pathway constructed in yeast (*Saccharomyces cerevisiae*) for the production of (*R*,*S*)-reticuline from the exogenously provided substrate, (*R*,*S*)-tetrahydropapaveroline (THP, also known as norlaudanosoline) [71]. 6OMT, norcoclaurine 6-*O*-methyltransferase; CNMT, coclaurine *N*-methyltransferase; 4′OMT, 3′-hydroxy-*N*-methylcoclaurine 4′-*O*-methyltransferase.

**Figure 13:**
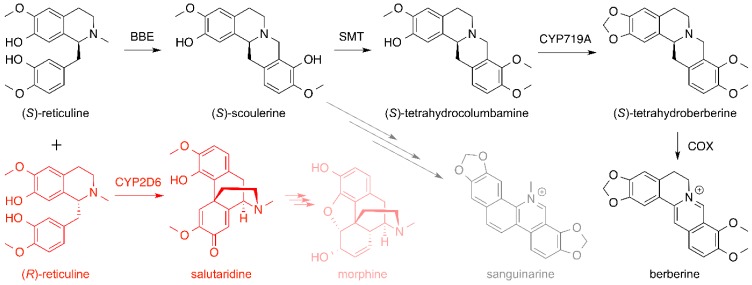
Extension of the *Saccharomyces cerevisiae* heterologous reticuline biosynthetic pathway for the production of precursors of the berberine- and sanguinarine-type benzylisoquinoline alkaloids (in black) and the morphinan alkaloids (in red). Compounds that are actually biosynthesized by the engineered yeast strains [71,72] are shown in solid, whereas downstream products are shown in semi-transparent. BBE, berberine bridge enzyme; SMT, (*S*)-scoulerine 9-*O*-methyltransferase; CYP719A, canadine synthase cytochrome P450 719A; COX, canadine oxidase; CYP2D6, human cytochrome P450 2D6.

**Figure 14:**
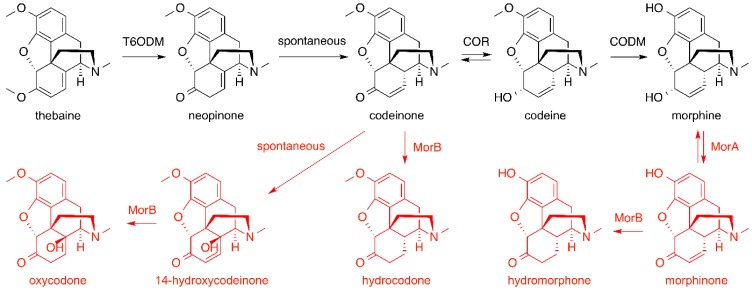
Heterologous biosynthetic pathway constructed in *Saccharomyces cerevisiae* for the production of morphinan alkaloids and related semi-synthetic opioids from thebaine, the exogenously provided starting material [73]. The biosynthetic pathway leading to the formation of morphine, the prominent member of the morphinan alkaloids, is shown in black. The biosynthetic pathways leading to the formation of semi-synthetic opioids, hydromorphone, hydrocodone and oxycodone, are shown in red. T6ODM, thebaine 6-*O*-demethylase; COR, codeinone reductase; CODM, codeine *O*-demethylase; MorA, morphine dehydrogenase; MorB, morphinone reductase.

**Figure 15:**
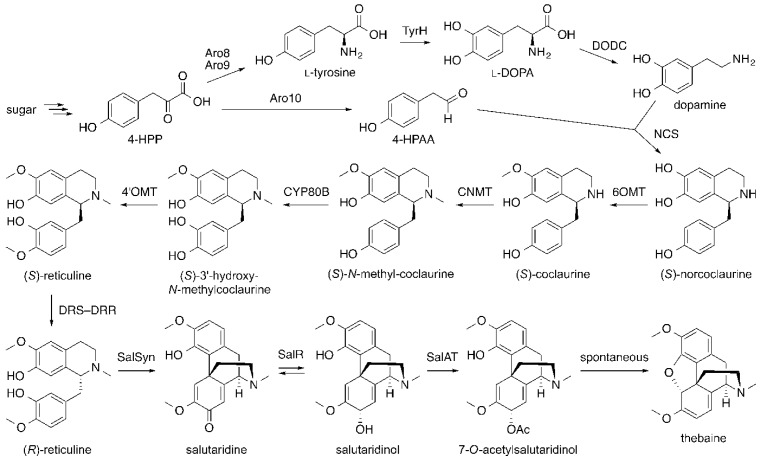
Heterologous biosynthetic pathway installed in *Saccharomyces cerevisiae* for de novo production of morphinan alkaloids and their semi-synthetic compounds from simple sugar [78]. Only the pathway leading to the formation of the key biosynthetic intermediate thebaine is shown. For the biosynthetic pathway leading to the formation of morphine and semi-synthetic morphinan opioids, see Figure 14. 4-HPP, 4-hydroxyphenylpyruvate; 4-HPAA, 4-hydroxyphenylacetaldehyde; l-DOPA, l-3,4-dihydroxyphenylalanine; Aro8, *S. cerevisiae* aromatic aminotransferase I; Aro9, *S. cerevisiae* aromatic aminotransferase II; TyrH, tyrosine hydroxylase; DODC, l-DOPA decarboxylase; Aro10, *S. cerevisiae* phenylpyruvate decarboxylase; NCS, norcoclaurine synthase; CYP80B, *N*-methylcoclaurine 3′-hydroxylase; DRS–DRR, 1,2-dehydroreticuline synthase–1,2-dehydroreticuline reductase; SalSyn, salutaridine synthase; SalR, salutaridine reductase; SalAT, salutaridinol 7-*O*-acetyltransferase.

The last section will look at opiates, which is a group of alkaloids that has been known to humans for about 6000 years and played a significant role as analgesic therapeutics and as abused recreational substances [66]. Opiates are naturally occurring members of opioids that can be isolated from the latex collected from opium poppy *Papaver somniferum*. The representative examples of opiates include reticuline, thebaine, codeine and morphine (Figure 12, Figure 13, Figure 14 and Figure 15). Opioids also include semi-synthetic and synthetic compounds, for example oxycodone and fentanyl, that interact with opioid receptors to effect physiological responses, such as analgesia, euphoria and respiratory depression [67]. Currently, the pharmaceutical industry relies heavily on plants as the predominant source of opiates and opiate-derived compounds. However, plant cultivation is labor intensive, difficult to scale up and hard to maintain yield consistency. While metabolic engineering of plants has been attempted to improve growth parameters and opiate productivity, the efforts have generally been met with difficulties thus far [68]. More recently, metabolic engineering of microbes has made a significant progress and is quickly becoming a viable alternative strategy for improving the opiate production process. Not only microbe cultivation is lower in cost and easier to scale up than plant cultivation, the product can be purified more easily from microbial extracts than from plant extracts due to lack of similar metabolites [47,69,70]. Below, we will discuss recent successes in developing microbe-based biosynthetic systems that produce a type of opiates called benzylisoquinoline alkaloids (BIAs).

In one approach toward developing microbes that can biosynthesize opiates, Smolke et al. engineered yeast *S. cerevisiae* to produce a series of BIAs. First, they developed a yeast strain harboring a set of biosynthetic genes that successfully produced (*R*,*S*)-reticuline upon feeding of a racemic mixture of tetrahydropapaveroline (THP), also known as norlaudanosoline (Figure 12) [71]. To produce reticuline, the key starting intermediate in the BIA biosynthetic pathway, a pair of plasmids carrying three biosynthetic genes encoding norcoclaurine 6-*O*-methyltransferase (6OMT), coclaurine-*N*-methyltransferase (CNMT) and 3′-hydroxy-*N*-methylcoclaurine 4′-*O*-methyltransferase (4′OMT) from *Thalictrum flavum* (yellow meadow-rue) or *P. somniferum* were introduced to *S. cerevisiae*. All three enzymes were known to accept both (*R*)-and (*S*)-enantiomers of their substrates [74,75]. The yeast strain carrying those three genes was able to biotransform the fed (*R*,*S*)-THP into (*R*,*S*)-reticuline (Figure 12). Then, a plasmid-born biosynthetic pathway comprised of the *P. somniferum* berberine bridge enzyme gene (*BBE*), the *T. flavum* (*S*)-scoulerine 9-*O*-methyltransferase gene (*SMT*) and the *T. flavum* canadine synthase gene (*CYP719A1*, a P450 gene) was established in the reticuline-producing yeast to produce BIA products found along the sanguinarine and berberine biosynthetic pathways. This yeast strain was able to produce (*S*)-scoulerine (sanguinarine precursor), (*S*)-tetrahydrocolumbamine and (*S*)-tetrahydroberberine (also known as canadine, a berberine precursor) (Figure 13, in black) [71]. Subsequently, incorporation of the canadine oxidase (COX) from *Berberis wilsonae* [76] and optimization of the berberine biosynthetic pathway in yeast led to successful production of berberine from fed (*R*,*S*)-THP [72]. Furthermore, a successful production of the first intermediate of the morphinan alkaloid biosynthesis pathway, salutaridine, was also accomplished by introducing a human P450 gene *CYP2D6* into the reticuline-producing yeast strain (Figure 13, in red). Related to this work, another group also established a biosynthetic pathway in yeast that converted fed (*R*,*S*)-THP into dihydrosanguinarine, an immediate precursor of sanguinarine [77], through the formation of (*R*,*S*)-reticuline and (*S*)-scoulerine using a biosynthetic scheme that was practically identical to that developed by Smolke et al. [71].

One unique strategy developed by Smolke et al. for optimizing the performance of a heterologous biosynthetic pathway in yeast was to use a GAL1-10 promoter system that is tunable with galactose concentration. Using this tunable expression system, Smolke et al. determined the optimal expression level of each of the heterologous genes for the production of desired products in yeast. Once the optimal expression level for each of the genes was determined, promoter of appropriate strength was selected for each of the genes to fine-tune the overall performance of the biosynthetic pathway. This type of optimization may be crucial in this type of study, where genes from diverse sources are assembled together to form a cooperative metabolic pathway. Streamlining the identification of the bottleneck in a newly installed heterologous metabolic pathway can also be accomplished by using a systems biological approach to illuminate the source of stress that is imposed on the host cell by expressing a foreign gene. In one example, effect of overproducing a foreign P450 in yeast was analyzed by DNA microarray analyses to reveal that transcription factors associated with iron starvation and heme-dependent gene regulation were overrepresented when the foreign P450 gene was expressed. This result indicated heme depletion as a possible stress in the system, and the activity of the target P450 was elevated by engineering the host′s heme biosynthesis to increase the intracellular heme level [79]. A similar approach can be taken to identify the source of stress in an engineered strain so that necessary measures can be implemented to eliminate the bottleneck in the exogenous biosynthetic pathway of interest.

The Smolke group also developed engineered yeast strains that can convert fed thebaine into codeine, morphine, hydromorphone, hydrocodone and oxycodone (Figure 14) [73]. To establish the biosynthetic pathway that converts thebaine into morphine, three *P. somniferum* genes, 6-*O*-demethylase (*T6ODM*), codeinone reductase isoform 1.3 (*COR1.3*) and codeine *O*-demethylase (*CODM*), were assembled into a single yeast artificial chromosome (YAC) and introduced into yeast. In one pathway, thebaine is converted into codeinone by T6ODM via the formation of neopinone. Then, COR reduces the carbonyl of codeinone to yield codeine. Finally, codeine is demethylated to morphine by CODM (Figure 14, in black). With the feeding of thebaine, the yeast strain carrying the YAC was able to produce codeine and morphine successfully. However, the pathway performance was improved by supplementing the culture medium with 2-oxoglutarate, a key co-substrate of dioxygenases T6ODM and CODM, as well as the precursors of 2-oxoglutarate, l-glutamine and l-glutamic acid. The copy number of the three genes was also optimized, where the ratio of 2:1:3 for *T6ODM*:*COR1.3*:*CODM* copy number was determined to give the best result with 5.2 mg/L of morphine being produced. The morphine-producing yeast was further engineered with two genes, morphine dehydrogenase (*morA*) and morphinone reductase (*morB*) from *Pseudomonas putida* M10 to biosynthetically prepare semi-synthetic morphine derivatives, hydromorphone, hydrocodone and oxycodone (Figure 14, in red).

Most recently, the yeast engineering effort was able to develop a strain capable of producing thebaine and hydrocodone from simple sugar [78]. To eliminate the need for supplementation of THP or thebaine, steps leading to the formation of (*S*)-reticuline were established by incorporating five plant-derived genes, including *6OMT*, *CNMT* and *4′OMT* from *P. somniferum* that were also used in the previous study [71]. In this system, dopamine, which is formed from l-tyrosine by the combined action of a mammalian and a bacterial enzyme, and 4-hydroxyphenylacetaldehyde (4-HPAA), an intermediate in the aromatic amine degradation pathway [80], were combined together by *Coptis japonica* (Japanese goldthread) norcoclaurine synthase (NCS) to form (*S*)-norcoclaurine (Figure 15). Compared to THP, norcoclaurine lacks one hydroxyl group. Thus, *CYP80B*, another gene from the *Eschscholzia californica* (California poppy) *N*-methylcoclaurine 3′-hydroxylase, also had to be included in the pathway to produce (*S*)-reticuline. Once (*S*)-reticuline became available, it was converted into (*R*)-reticuline by introducing 1,2-dehydroreticuline synthase–1,2-dehydroreticuline reductase (DRS–DRR), a natural fusion protein from *Papaver bracteatum* (Iranian poppy), into the system to initiate the morphinan alkaloid biosynthesis. Unlike the previous study that used the human P450 CYP2D6 to convert (*R*)-reticuline into salutaridine (Figure 13, in red) [71], this study employed an engineered *P. bracteatum* salutaridine synthase (SalSyn) that was fused to another plant P450 cheilanthifoline synthase to drive proper folding and glycosylation of *P. bracteatum* SalSyn in yeast. This fusion enzyme attained an approximately six-fold greater activity than the native *P. bracteatum* SalSyn [78]. Subsequently, salutaridine was reduced by *P. bracteatum* salutaridine reductase (SalR) to yield salutaridinol, which was then acetylated by *P. somniferum* salutaridinol 7-*O*-acetyltransferase (SalAT) to form 7-*O*-acetylsalutaridinol. Finally, 7-*O*-acetylsalutaridinol was spontaneously converted into thebaine, completing the de novo biosynthesis of the key intermediate for morphine biosynthesis in yeast. Conversion of thebaine to hydrocodone was achieved by essentially the same approach used in the previous study (Figure 14) [73]. Overall, the complete biosynthesis of hydrocodone from simple sugar required introduction and engineering of a total of 23 genes from plants (11 genes from four plant species, including one extensively engineered SalSyn gene), mammal (one engineered gene from rat), bacteria (two genes from *P. putida*) and yeast (nine genes, of which four were modified). 

**Figure 16:**
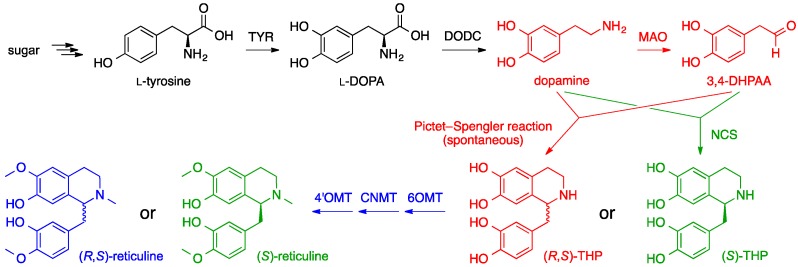
Heterologous biosynthetic pathway established in *E. coli* for de novo production of (*R*,*S*)-reticuline from simple sugar using a three-step fermentation method [81,82,83]. The three fermentation steps are designated by three different colors: black for the dopamine production step, red for the (*R*,*S*)-THP production step and blue for the (*R*,*S*)-reticuline production step. The initial attempt to use the norcoclaurine synthase (NCS) for the production of (*S*)-THP for the preparation of (*S*)-reticuline (shown in green) was replaced by the use of a spontaneous intracellular Pictet-Spengler reaction (shown in red) that resulted in the eventual formation of (*R*,*S*)-reticuline (shown in blue). Subsequent biosynthesis of thebaine and hydrocodone followed the same biosynthetic scheme shown in Figure 14 and Figure 15, respectively. MAO, monoamine oxidase.

In another approach, Sato et al. focused their efforts on establishing a BIA biosynthetic pathway in *E. coli* and successfully developed production systems capable of biosynthesizing (*R*,*S*)-THP, (*R*,*S*)-reticuline, thebaine and hydrocodone (Figure 16) [70,81,82,83]. Their strategy relied on supplying the key intermediate reticuline as a racemic mixture using an artificial biosynthetic pathway comprised of tyrosinase, l-3,4-dihydroxyphenylalanine (l-DOPA) decarboxylase and monoamine oxidase (MAO) obtained from various bacterial sources. These three enzymes work together to convert l-tyrosine into dopamine and 3,4-dihydroxyphenylacetaldehyde (3,4-DHPAA) [81]. Initially, an NCS gene was introduced to the system to combine dopamine and 3,4-DHPAA into (*S*)-THP for subsequent conversion into (*S*)-reticuline [70,81]. However, NCS was later eliminated to rely on the spontaneous formation of a racemic mixture of THP from dopamine and 3,4-DHPAA via a Pictet-Spengler reaction inside the cells [82]. Then, (*R*,*S*)-THP was converted into (*R*,*S*)-reticuline by 6OMT, CNMT and 4′OMT [83]. This was the same set of three enzymes that was used by Smolke et al. to prepare (*S*)-reticuline from (*R*,*S*)-THP (Figure 12) [71], except all of the genes used by Sato et al. originated from *C. japonica*. Once *E. coli* strains for the production of reticuline were established, *P. somniferum* genes that code for SalR, SalAT and engineered SalSyn having its *N*-terminal transmembrane region removed were added to the system to produce thebaine [83]. Lastly, addition of *P. somniferum T6ODM* and *P. putida morB* genes accomplished the biosynthesis of hydrocodone [83]. This *E. coli*-based biosynthetic system developed by Sato et al. employed a stepwise fermentation technique to avoid tyrosinase from degrading the early intermediate THP [82]. This method also helped minimize product inhibition of pathway enzymes and propagation of undesired byproducts through the biosynthetic steps. Those factors are thought to have contributed to the superior product yield obtained with their multi-step *E. coli* fermentation strategy for the opioid biosynthesis.

## 7. Conclusions

Over the past several decades, elucidation of metabolic pathways leading to the biosynthesis of various alkaloids has seen a tremendous advancement. Those efforts have been facilitated greatly by the rapid accumulation of genomic sequence information and the use of heterologous host organisms that allow efficient reconstitution of the biosynthetic pathway being investigated. For the heterologous host, *E. coli*, *S. cerevisiae* and *A. nidulans* are used most commonly. Those hosts are particularly attractive because of their fast and robust growth and ease of genetic manipulation. They also have a metabolic background that imposes low interference to the alkaloid biosynthesis being studied. In addition to those in vivo analyses, in vitro characterizations of the biosynthetic enzymes also provided deep insight into the mechanisms involved in the generation of representative members of alkaloids. Biosynthesis of some of those compounds, such as diketopiperazine and tetrahydroisoquinoline natural products, employ the use of NRPSs, while a great number of alkaloids rely on oxidoreductases, primarily cytochrome P450s and flavin-containing monooxygenases, for extensive modifications of the core scaffolds that are typically constructed from aromatic amino acids and isoprenes. Combining the current efforts in the discovery of alkaloid biosynthetic pathways with the knowledge and experiences acquired from previous successful incorporation and engineering of NRPSs, oxidoreductases and other auxiliary enzymes in bacteria, yeast and filamentous fungi could further generalize our approach for comprehensive characterizations of alkaloid biosynthetic pathways and mass-production of clinically and industrially valuable alkaloids that are typically produced in limited quantity by the original producers. Detailed knowledge of biosynthesis of those compounds will also facilitate application of synthetic biological approaches in developing efficient and sustainable platforms for preparing semi-synthetic analogs of alkaloids.
